# 3D-Printed Quasi-Cylindrical Bragg Reflector to Boost the Gain and Directivity of cm- and mm-Wave Antennas

**DOI:** 10.3390/s21238014

**Published:** 2021-11-30

**Authors:** Jéssica A. P. Ribeiro, Hugo R. D. Filgueiras, Arismar Cerqueira Sodré Junior, Felipe Beltrán-Mejía, Jorge Ricardo Mejía-Salazar

**Affiliations:** 1 National Institute of Telecommunications (Inatel), Santa Rita do Sapucaí 37540-000, MG, Brazil; jessica.ribeiro@mtel.inatel.br (J.A.P.R.); hugo.rodrigues@inatel.br (H.R.D.F.); arismar@inatel.br (A.C.S.J.); 2 Padtec, Campinas 13098-396, SP, Brazil; fmejia@padtec.com.br

**Keywords:** antennas, Bragg reflectors, diffraction, directivity, gain, multilayer structures, radiation pattern, WiFi

## Abstract

We demonstrate a concept for a large enhancement of the directivity and gain of readily available cm- and mm-wave antennas, i.e., without altering any property of the antenna design. Our concept exploits the high reflectivity of a Bragg reflector composed of three bilayers made of transparent materials. The cavity has a triangular aperture in order to resemble the idea of a horn-like, highly directive antenna. Importantly, we report gain enhancements of more than 400% in relation to the gain of the antenna without the Bragg structure, accompanied by a highly directive radiation pattern. The proposed structure is cost-effective and easy to fabricate with 3D-printing. Our results are presented for frequencies within the conventional WiFi frequencies, based on IEEE 802.11 standards, thus, enabling easily implementation by non-experts and needing only to be placed around the antenna to improve the directivity and gain of the signal.

## 1. Introduction

Centimeter (cm)- and millimeter (mm)-wave antennas have attracted extensive research attention during recent decades due to their vast amount of indoor and outdoor applications. In particular, these antennas provide high data rates for broadband wireless communications [1], imaging [2], satellite communications [3] and collision avoidance devices [4]. Despite these advantages, cm- and mm-waves suffer severe detrimental effects on the quality of signal transmission due to path loss and blockages [5].

Although the use of antenna array designs seems to be the most natural way to overcome these drawbacks, such a mechanism could be expensive or complex due to the requirement of a feeding network with precise geometric positioning of radiating elements. Moreover, the large number of feeding elements in an array may also lead to transmission loss effects.

The use of metamaterials [6,7], metasurfaces [8,9,10] and multilayer lenses [11,12,13,14] have emerged as promising alternatives for the design and development of directive high-gain antennas. All these approaches are based on the use of artificial flat subwavelength structures to convert the radiated quasi-spherical waves into near-plane waves for gain enhancement. The main advantage of using these lenses is in the simplicity for design and fabrication. However, the implementation of these lenses is hampered by the relative large sizes and restrictive operation bandwidths, making the lens antennas bulky and inefficient.

On the other hand, photonic superlattices, i.e., alternating layers of materials with different refractive indexes, have become essential components of optoelectronic and microwave devices due to their ability to tailor and tune the electromagnetic wave propagation properties [15,16,17,18]. Particularly important are the quarter-wavelength Bragg mirrors, commonly called quarter-wave-stacks (QWSs), consisting of alternating layers with thicknesses (for each material) of one-quarter of the working wavelength [19,20], which completely reflect the incident electromagnetic waves.

Although these reflectors can be implemented to develop cavity enhanced antenna-gain [21], less attention has been paid to the use of Bragg mirrors to enhance the directivity and gain of readily available antennas. This latter application could be used, for example, to customize the indoor wireless coverage, which will not only improve the signal reception in desired regions but also increase the security by preventing information theft and interception techniques.

**Figure 1:**
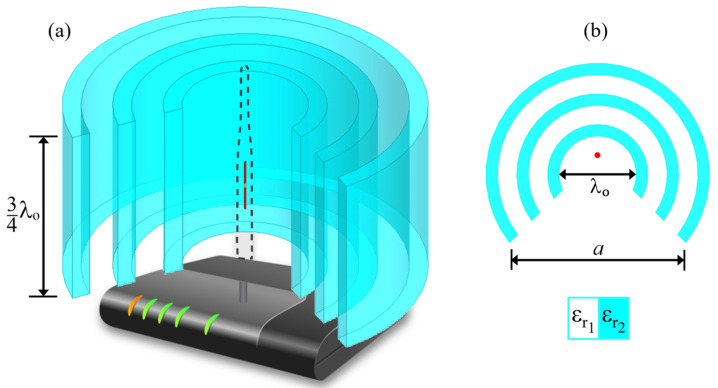
(**a**) Schematic representation of the proposed three layer dielectric structure for applications in 802.11 based wireless networks. The geometry is based on the H-plane radiation of AP antennas. (**b**) A top view of the quasi-cylindrical Bragg reflector with a gradual aperture.

In this work, we show that quasi-cylindrical Bragg reflector structures can be placed around a dipole antenna to produce directional radiation patterns. Our concept can be used, for example, placed around the half-wave dipole antenna of a conventional router–access point (AP)–in a wireless local area network (WLAN), as illustrated in Figure 1a, where a gradual aperture was used for directional radiation. The geometry of the structure was designed with two main aims.

First, the idea to produce a highly directive pattern, for which we used a patterned aperture in order to diffract the electromagnetic radiation to a specific region of the space. Second, inspired by the horn antenna design, we used a triangular aperture in order to provide a gradual transition from the Bragg structure to the free space, which, as observed from results here, enables an efficient and highly directive radiation mechanism. In Figure 1b, we show the cross section of the Bragg reflector, placed along the H-plane of the antenna.

Although our concept can be scaled to tune the working wavelength according to the requirements, we focused our attention here on a system working at the Unlicensed National Information Infrastructure (U-NII) frequency band, from 5.15 to 5.825 GHz, defined by the IEEE 802.11 standard [22]. For simplicity, we considered the structure built by alternating layers of air, with permittivity $\varepsilon_{r_{1}}=1.0$, and a plastic dielectric material with $\varepsilon_{r_{2}}$. The quasi-cylindrical Bragg reflector was manufactured by 3D printing technology [23,24] and used for beam steering the radiation pattern of a dipole antenna, improving the gain and enabling at will manipulation of directivity (while maintaining the bandwidth).

The paper is organized as follows. In Section 2, we show the design of a cylindrical structure comprising three mirror bilayers with a gradual aperture, resembling the radiation pattern of directive conical horn as a model. The measured and simulated results are discussed in Section 3. Finally, our conclusions are presented in Section 4.

## 2. Theoretical Framework

We consider a finite quasi-cylindrical Bragg reflector, with a gradual aperture, made by alternating layers of two different dielectric materials (see Figure 1). Then, we demonstrate that the radiation pattern profile of an AP antenna can be tuned, with boosted gain and directivity, without changing any parameters of the antenna design. The building layers were taken as made of air, with $\varepsilon_{r_{1}}=1.0$, and a plastic material, with $\varepsilon_{r_{2}}=2.9$ [25]. The layers have thicknesses $d_{i}=\frac{\lambda_{0}}{4\sqrt{\varepsilon_{r_{i}}}}$, equal to one quarter of the working wavelength at each medium.

**Figure 2:**
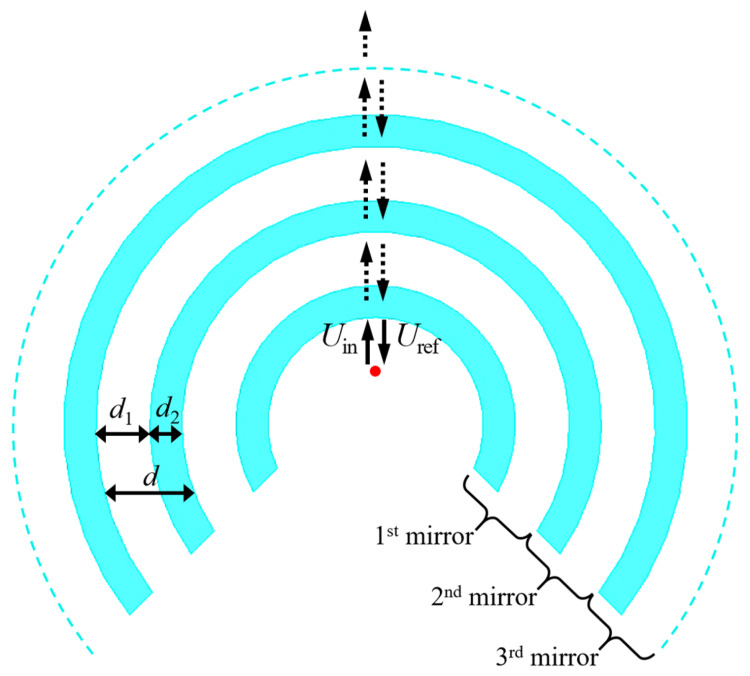
Illustration of the proposed quarter wavelength stack mirror. The red dot represents a half-wave dipole antenna as seen from above. The light-blue curves represent the quasi-cylindrical dielectric layers, and the white background represents air.

Although the reflection amplitudes of multilayer structures increase with the number of bilayers in the system, we considered only three bilayers to show the simplicity of our concept. For the analytical treatment, we labeled each bilayer (from the center) as mirror 1, 2 and 3, as shown in Figure 2, with bilayer thicknesses $d=d_{1}+d_{2}$. Considering the AP antenna placed at the center of the structure, and, keeping in mind that conventional AP dipole antennas exhibit omnidirectional radiation patterns along the H-plane, the corresponding electromagnetic field impinges almost normally along the entire interface.

Since the radiated field can only reach the external region through the gradual aperture, the quasi-cylindrical Bragg reflector works as an electromagnetic cavity with an aperture, imposing high directivity to the corresponding radiation pattern.

Let us start by giving an analytical description of the device here presented. In Figure 2, we plotted a top view of the mirror design, where we indicate the corresponding layer ($d_{i}$) and bilayer (*d*) thicknesses. The position of the AP antenna is also indicated by a red dot at the center, whilst the incident ($U_{\mathrm{in}}$) and reflected ($U_{\mathrm{ref}}$) electromagnetic fields inside the cavity are represented by upward and downward arrows, respectively.

The electromagnetic fields in the system can be represented as functions of the reflection (*R*) and transmission (*T*) coefficients. Since the building materials are considered transparent for microwave radiation, i.e., with almost lossless transmission, the reflective features of the system are completely due to cumulative constructive interference effects of the electromagnetic waves along the structure [26]. For the system in Figure 2, we can represent $U_{\mathrm{ref}}$ as

(1) $$U_{\mathrm{ref}}=RU_{\mathrm{in}}+\left(RTU_{\mathrm{in}}\;e^{-i2\beta d}\right)+\left(RT^{2}U_{\mathrm{in}}\;e^{-i4\beta d}\right)T^{2},$$

with $\beta =2\pi /\lambda_{\circ }$, where $\lambda_{\circ }$ is the vacuum wavelength. Equation (1) was obtained by the sum of the contributions from all the partial reflections throughout the structure, i.e., $RU_{\mathrm{in}}$ represents the reflection contribution from the inner mirror, $RTU_{\mathrm{in}}e^{-2i\beta d}$ is the reflection contribution from the second (at the middle) mirror, and $RT^{2}U_{\mathrm{in}}e^{-i4\beta d}$ is the reflection contribution from the outer mirror.

Therefore, the corresponding reflection coefficient for the system is given by,

(2) $$R_{f}=\frac{U_{\mathrm{ref}}}{U_{\mathrm{in}}}=R\left(1+T^{2}\;e^{-i2\beta d}+T^{4}e^{-i4\beta d}\right).$$

Multiplying both sides of (2) by ($1-T^{2}\;e^{-i2\beta d}$), and, after a few steps of algebra, we reach

(3) $$R_{f}=R\frac{1-{\left(T^{2}\;e^{-i2\beta d}\right)}^{3}}{1-(T^{2}\;e^{-i2\beta d})},$$

with modulus

(4) $$\left|R_{f}\right|=\left|R\right|\sqrt{\frac{1+\left|T^{12}\right|-2{\left|T\right|}^{6}\;\cos\left[6\left(\beta d-\phi \right)\right]}{1+\left|T^{4}\right|-2{\left|T\right|}^{2}\;\cos\left[2\left(\beta d-\phi \right)\right]}},$$

where ϕ is the phase angle. In order to represent (4) only in terms of *R*, we use the relation for the electromagnetic energy conservation (${\left|T\right|}^{2}+{\left|R\right|}^{2}=1$), and then we obtain

(5) $$|R_{f}\left|=|R\right|\sqrt{\frac{1+|{1-{\left|R\right|}^{2}|}^{6}-2|1-{{\left|R\right|}^{2}|}^{3}\;\cos\left[6\left(\beta d-\phi \right)\right]}{1+{{\left|1-|R\right|}^{2}|}^{2}{-2|1-|R|}^{2}\;\cos\left[2\left(\beta d-\phi \right)\right]}}.$$

By varying the quantity βd−ϕ, inside (5), we can produce sets of maximum and minimum values of $\left|R_{f}\right|$. In particular, for βd−ϕ=mπ, with *m* as a positive integer number, we have maximum (minimum) values for the reflection (transmission) coefficient, which indicate constructive (destructive) interference for the reflected (transmitted) fields along the structure. These results show how to tune and tailor the thicknesses based on the optical properties of the elementary components of our design, as will be shown below.

### 2.1. Layers Design for High Reflectivity on H-Plane

Using (5) and the condition for maximum $\left|R_{f}\right|$ (βd−ϕ=mπ), we obtain

(6) $$d=m\frac{\lambda_{\circ }}{2}$$

for the mirror’s thicknesses. Then, we have $d_{1}=\left(1/4\right)\left(\lambda_{\circ }/n_{1}\right)$ and $d_{2}=\left(1/4\right)\left(\lambda_{\circ }/n_{2}\right)$ for the elementary building layers, with $n_{1}=\sqrt{\varepsilon_{r_{1}}}$ and $n_{2}=\sqrt{\varepsilon_{r_{2}}}$ for the corresponding refractive indices. Our results are presented for the structure, projected to work at f=5.7 GHz with the corresponding working wavelength $\lambda_{\circ }=52$ mm.

### 2.2. Gain Analysis for Triangular Aperture Optimization

The proposed model has a triangular shaped aperture to modify the radiation pattern in order to reach performances similar to that of horn antenna designs. This aperture starts from a vertex with a distance $\lambda_{\circ }/4$ from the first mirror, exactly where the AP antenna is placed. The total aperture *a* is the triangle base shown in Figure 1b. The optimum aperture dimension of the aperture was selected in order to optimize the corresponding gain and directivity of the antenna. The results were obtained using the commercial ANSYS HFSS^®^ software, which implements the Finite Element Method. For all the analyses, the radiator was centered in the model at x=y=z=0, with the layered structure around it.

**Figure 3:**
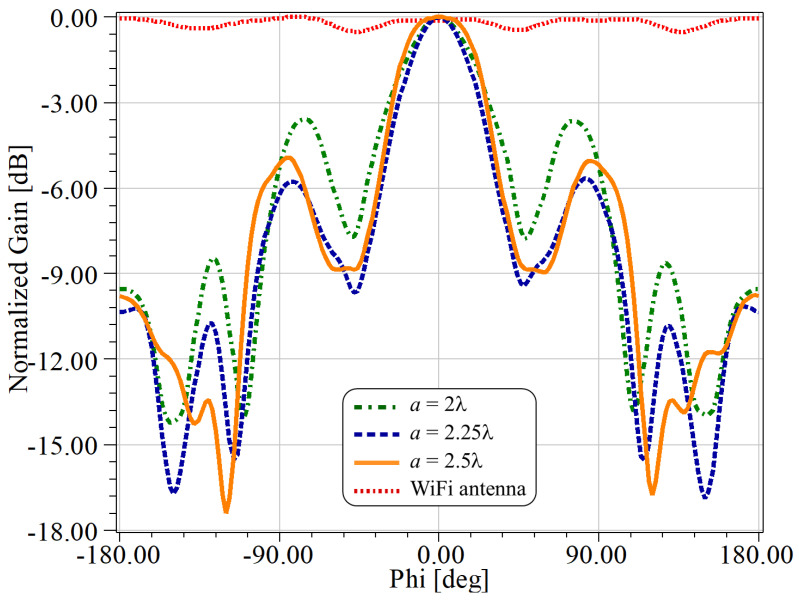
Comparative results for the antenna with (orange, blue and green lines) and without (red-dotted line) the Bragg reflector structure. Calculations were made for different *a*–values considering f=5.7 GHz, with $-180^{\circ }<\phi <+180^{\circ }$, along the H-plane ($\theta =90^{\circ }$). a=2.25λ produced the lowest side lobes.

In order to choose the best value for *a*, we made a parametric optimization using the radiation pattern of the antenna and an aperture range of $2\lambda_{\circ }\leq a\leq 2.5\lambda_{\circ }$. The results are shown in Figure 3 for f=5.7 GHz, where we show a comparison of the normalized radiation patterns along the H-plane for the antenna with and without the multilayer structure. More specifically, we considered the results for a=104, 117 and 130 mm, which are, respectively, a=2λ, 2.25λ and 2.5λ.

From these results, we clearly observe an improvement in the directivity of the antenna when using the proposed multilayer structure. It can also be noted that the main lobe is found at $\phi =0^{\circ }$ for $\theta =90^{\circ }$, which was found to be insensitive to changes on *a* at 5.7 GHz. Moreover, these results indicate that the side lobe amplitudes can also be tuned through the proper selection of the value of *a*. Indeed, the best relationship between the main lobe and the largest side lobes (about 6 dB at $\phi =-87^{\circ }$ and at $\phi =+87^{\circ }$) was obtained for an aperture of a=117 mm, as shown by the dashed blue line in Figure 3.

## 3. Results and Discussion

**Figure 4:**
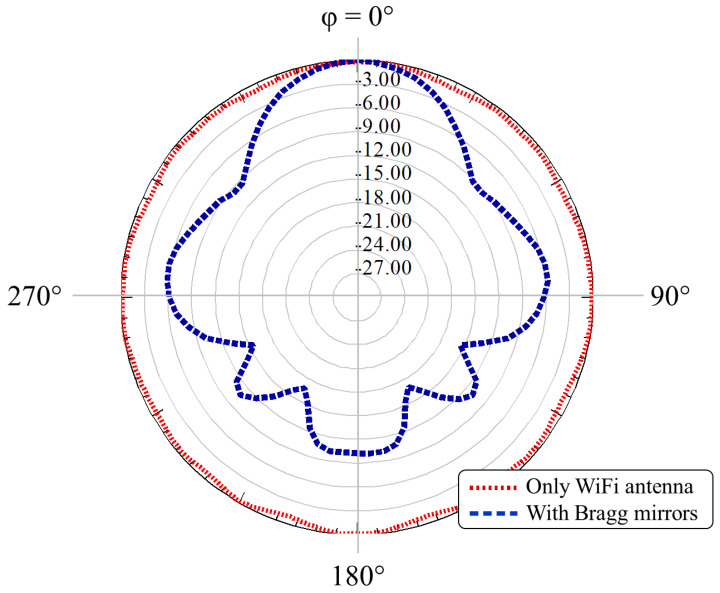
Normalized radiation pattern at f=5.7 GHz, for $0^{\circ }<\phi <360^{\circ }$ and $\theta =90^{\circ }$ (H-plane). The figure compares the radiation pattern with (dashed blue curve) and without (dotted red curve) the all-dielectric Bragg reflector.

**Figure 5:**
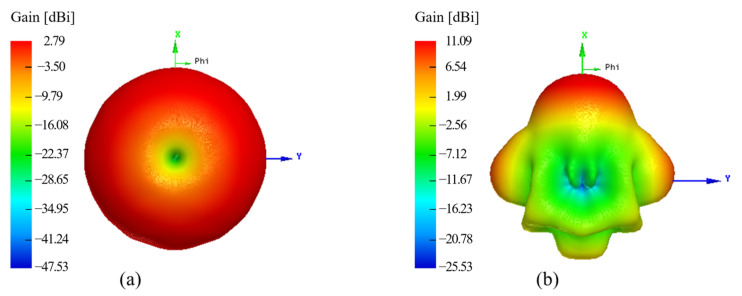
Comparative numerical results for the gain (**a**) of the dipole antenna and (**b**) the antenna with the quasi-cylindrical Bragg structure.

Figure 4 shows a comparison of the radiation patterns, using the working frequency f=5.7 GHz, for the antenna with (dashed line) and without (dotted line) the Bragg reflector. In addition, a top view of the gain for the system without the multilayer structure is presented in Figure 5a, whilst the case with a Bragg reflector is shown in Figure 5b. In contrast to the antenna without reflectors, which only exhibited gains of around 2 dBi, we note gain enhancements higher than 8 dB when considering the reflector.

**Figure 6:**
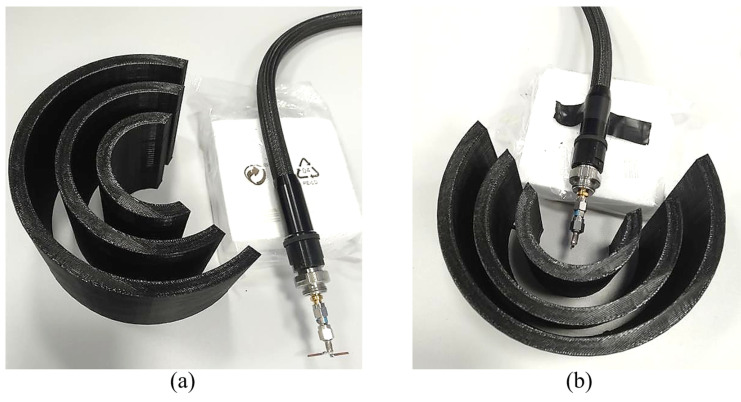
(**a**) The dipole antenna and the Bragg quasi-cylindrical structure and (**b**) the Bragg reflector around the dipole antenna.

In order to experimentally demonstrate the feasibility of our concept, we used a 3D printing technique with PLA (polylactic acid) material ($\varepsilon_{r\mathrm{PLA}}=2.9$) to manufacture the structure for a working wavelength λ=52 mm. More specifically, we used a 3D-printer CL2 Pro Plus from Cliever (Brazil). The structure was fabricated in a time of approximately 51 h. Figure 6 shows pictures from two different perspectives of the complete 3D printed quasi-cylindrical Bragg reflector. The PLA is a biodegradable polymer, produced from renewable resources, which has gained considerable interest in 3D printing during the last years.

In particular, we used a commercial filament PLA Ingeo 4043D from NatureWorks, which has considerable 3D printing features, such as precise detail and less warping or curling, as well as good impact resistance in the printed parts (see Ref. [27] for details). The dimensions of the fabricated structure are $d_{1}=13$ mm, $d_{2}=7.95$ mm and a=117 mm. It has 1.5λ height and 2.5λ width.

**Figure 7:**
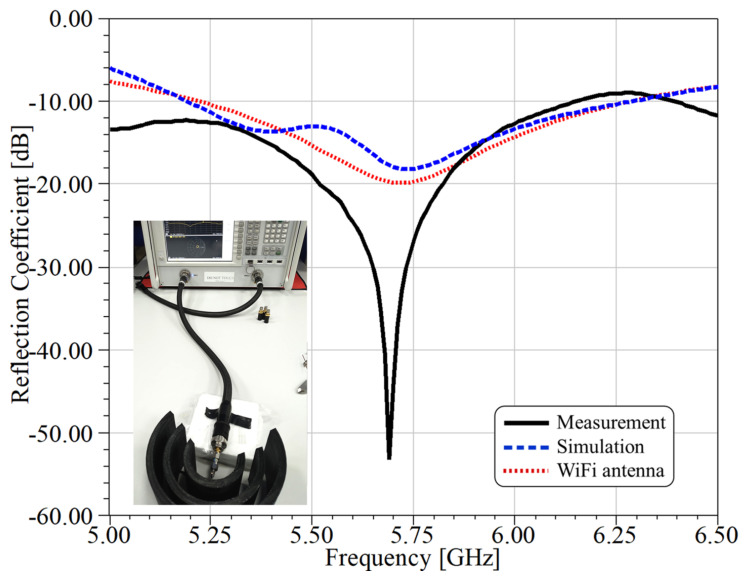
The experimental results for the $S_{11}$ parameter for the antenna with (solid–black curve) and without (blue-dashed line) the Bragg reflector. The red dotted curve corresponds to simulations for the antenna without the Bragg reflector.

The reflection coefficient ($S_{11}$ parameter) was measured (solid black curve) and compared with the simulation (dashed blue curve) results, shown in Figure 7. The picture inside this figure shows the setup for the reflection coefficient measurement of the proposed dielectric structure with dipole feeding. The PNA (programmable network access) vector network analyzer N5224A (from 100 MHz to 43.5 GHz) from Keysight was used. In contrast to the numerical results for the antenna without a reflector (see the dotted red curve), we observed an improvement of around 35 dB in the experimental measurements when using the reflector.

Simulations and measurements were carried out for a half wave dipole antenna to verify that the use of the Bragg reflector did not influence the resonance point. Moreover, measurements demonstrated bandwidth improvements ($S_{11}<-10$ dB) when the Bragg reflector structure was considered (see Figure 7). Small differences were observed for the experimental and numerical results, which could be due to small misalignments in the period length of the Bragg structure and possible differences between the nominal and real permittivity values for the PLA. We also consider that the dipole antenna was not perfectly placed at the center of the structure, as assumed in the numerical simulations.

**Figure 8:**
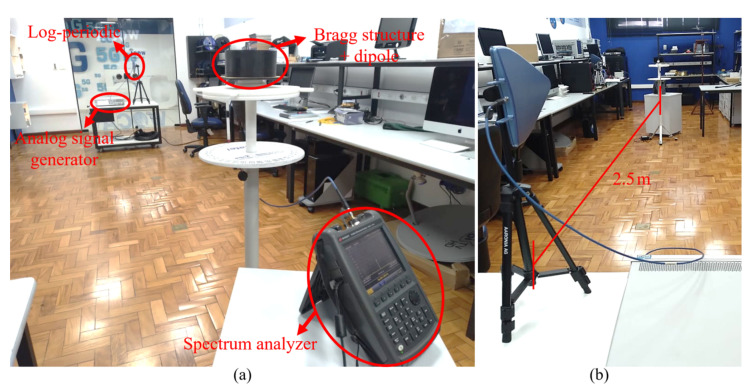
Pictures of the experimental setup taken (**a**) from the Bragg reflector to the antenna and (**b**) from the antenna to the Bragg reflector.

Pictures of the experimental setup used for the measurements are shown in Figure 8a,b, where a 5 dBi gain log periodic antenna (HyperLOG 60100 from Aaronia AG) was used as reference and an analog signal generator (Keysight EXG N5172B (9 kHz–6 GHz)) was used for transmission. For the reception side, we considered the proposed dielectric Bragg reflector structure, around a half wave dipole antenna and a spectrum analyzer (Keysight FieldFox N9952A (50 GHz)). It was guaranteed that the transmitting and receiving antennas were at the same height and at the far-field distance between the transmission and reception.

**Figure 9:**
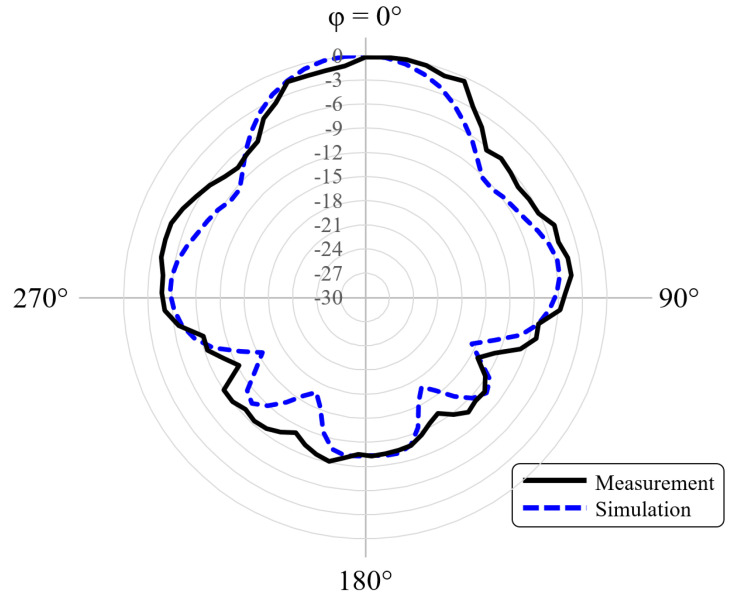
The comparative results for the measured (solid-black curve) and simulated (dashed-blue line) radiation patterns at a 5.7 GHz operating frequency. The results in this figure consider the use of the proposed quasi–cylindrical Bragg structure.

Since the experiments were made in a realistic environment (not in an anechoic chamber), very low transmission power was used to mitigate reflections. Indeed, the radiation pattern was measured by sweeping the azimuth to verify the maximum received power. The normalized radiation patterns for the H-plane at 5.7 GHz are shown in Figure 9, from which we observe very good agreement between the experiment and simulations, including the null points feature. The measurements exhibited a 9.8 dBi gain for the WiFi dipole antenna when using the Bragg structure, which is also in very good agreement with the numerical simulations.

## 4. Comparison with Previous Works

In Table 1, we summarize the main reflecting performance of our concept in comparison with some recent proposals. In particular, we compared our all-dielectric Bragg reflector with the use of large parabolic metallic reflectors [28], expensive and more complex metamaterial frequency selective surfaces (FSS) [29,30] and metallic covered 3D printed designs [31,32].

## 5. Conclusions

In summary, we numerically demonstrated and experimentally fabricated a quasi-cylindrical Bragg reflector, with a triangular aperture, used to boost the gain and directivity of readily available cm- and mm-wave antennas.

In particular, we focused on a frequency f=5.7 GHz to elucidate the working principle of our proposal, which can be extended to higher and lower frequency regimes. Significantly, we used only a multilayer composed of three very simple bilayers, which can be easily fabricated using household 3D-printing technology. Directivity enhancements were accompanied by gain enhancements of more than 400% in relation to the gain for the antenna without the Bragg structure.

## Figures and Tables

**Table 1:** Comparison of the performance of the proposed reflector with other structures found in the literature.

Reference	Radiation		Manufacturing		
	WiFi Frequency	Gain Improvement	Size	Fabricated	Installation Local
[28]	2.4 GHz	10 dB	1.2λ×3.2λ	Parabolic reflector with aluminum and wood	Close to antenna
[29]	5.5 GHz	10 dB	λ×1.21λ (each array)	Corner Reflector with FSS	Close to antenna
[30]	5.8 GHz	9 dB	1.16λ×1.74λ (each array)	Corner reflector with FSS	Close to antenna
[31]	2.4 or 5 GHz		Different shapes	Dielectric 3D printed covered by aluminum	Close to antenna
[32]	2.4 GHz	6 dB	Different shapes, according to the signal	Dielectric 3D printed covered by aluminum	Close to antenna
Proposed Bragg structure	5 GHz	9 dB	1.5λ×2.5λ	All dielectric 3D printed	Close to antenna

## Data Availability

Data underlying the results presented in this paper are not publicly available at this time but may be obtained from the authors upon reasonable request.
